# Cancer Cachexia: New Insights and Future Directions

**DOI:** 10.3390/cancers15235590

**Published:** 2023-11-26

**Authors:** Claudia Raluca Mariean, Oana Mirela Tiucă, Alexandru Mariean, Ovidiu Simion Cotoi

**Affiliations:** 1 Doctoral School of Medicine and Pharmacy, University of Medicine, Pharmacy, Science, and Technology George Emil Palade of Targu Mures, 540142 Targu Mures, Romania; 2 Pathophysiology Department, University of Medicine, Pharmacy, Science, and Technology George Emil Palade of Targu Mures, 540142 Targu Mures, Romania; 3 Dermatology Department, University of Medicine, Pharmacy, Science, and Technology George Emil Palade of Targu Mures, 540142 Targu Mures, Romania; 4 Dermatology Clinic, Mures Clinical County Hospital, 540342 Targu Mures, Romania; 5 Pulmonology Clinic, Mures Clinical County Hospital, 540103 Targu Mures, Romania; 6 Pathology Department, Mures Clinical County Hospital, 540011 Targu Mures, Romania

**Keywords:** cancer, cachexia, sarcopenia, inflammation, biomarkers, clinical impact, pathophysiology, CT-imaging

## Abstract

**Simple Summary:**

Cancer is a major burden of disease worldwide, with cancer cachexia being a poor prognosis factor which leads to a decreased quality of life and overall survival. There is an increased need for further studies regarding this complex phenomenon of metabolic imbalances, weight loss, inflammatory changes, and other important pathophysiological components that have a drastic impact on the prognosis of cancer patients. The main goal of this paper is to emphasize the importance of the early detection of cancer patients affected by cachexia and to provide a scientific update regarding the main pathophysiological mechanism involved in cancer cachexia pathogenesis and the complex clinical impact that this pathology has. We aim to raise awareness about the importance of identifying potential biomarkers for the early diagnosis of cancer cachexia, as a key factor in improving patients’ management and approach.

**Abstract:**

Cancer remains a major health problem and is associated with cachexia in up to 80% of cases, leading to decreased survival and quality of life. Cachexia involves complex metabolic disturbances in both protein and energy balance, muscle wasting phenomena, weight loss, systemic inflammation, overall decreased performance status, and tolerability to treatment. The clinical impact of cancer cachexia is very complex, with early detection of cachectic patients and identification of predictive biomarkers being two key factors for improving survival. Thus, a better understanding of the complexity of cancer cachexia phenomena and its main pathophysiological mechanism is much needed. Our review highlights the most important information about cancer cachexia, aiming to disseminate updated research findings about this highly deadly condition.

## 1. Introduction

Cancer is the leading cause of death in people aged 40–79 years old and is associated with cachexia in up to 80% of patients, depending on the cancer type [1,2]. At least 20% of cancer patients will die due to cancer cachexia [3]. Cancer patients need to be periodically assessed during treatment, as their nutritional status is continuously evolving depending on tumor stage, associated comorbidities, treatment type, and setting [4].

Cachexia was first described by Hippocrates as: “The flesh is consumed and becomes water…the abdomen fills with water, the feet and legs swell, the shoulders, clavicles, chest, and thighs melt away… The illness is fatal” [3]. It involves the disruption of homeostatic processes in both protein and energy balance, occurring in 50% of cancer patients, according to Han et al. Systemic inflammation and exacerbation of catabolic processes are usually associated [5].

Cancer induces metabolic disturbances, leading to sympathetic activation, decreased gonadal function, insulin resistance, and tumor-induced systemic inflammation. All of these changes coupled with poor food intake lead to muscle wasting, decreased performance status and tolerability to treatment, and in the end, death of the patient [2].

Cachexia leads to various body composition changes which increase treatment-related toxicity in cancer patients. Treatment doses are usually calculated based on body surface area but do not take into consideration muscle mass which is known to be decreased in sarcopenic and cachectic patients [3].

The main objective of this paper is to provide an update regarding the complex phenomena of cancer-related cachexia. There is unequivocal evidence that the relationship between cancer and cachexia should be further studied in order to develop adequate prognostic and therapeutic strategies to improve overall survival, quality of life, and treatment response rate [5].

## 2. Definition of Cachexia

Several definitions have been discussed in recent years for an appropriate description of cachexia. In 2008, the proposed definition of cachexia was: “Cachexia is a complex metabolic syndrome associated with an underlying illness and characterized by loss of muscle with or without loss of fat mass”. The key part of this definition was at least 5% bodyweight loss in the last 12 months or less (corrected for fluid retention). Cancer patients usually have shorter timeframes, varying from 3 to 6 months. A body mass index (BMI) less than <20.0 kg/m^2^ without a known history of bodyweight evolution was also considered diagnostic for cachexia, according to Evans et al. [3,6].

In the same year, a set of diagnostic criteria for cachexia was proposed. It was based on an unintentional weight loss of at least 5% in the last 12 months or less in the presence of an underlying disease, plus three of the following [3,6]:

- Fatigue
- Anorexia
- Abnormal biochemical lab results (Increased inflammatory markers: C reactive protein (CRP) > 5.0 mg/L, interleukin 6 (IL-6) > 4.0 pg/mL); anemia (<12 g/dL); low serum albumin (<3.2 g/dL)
- Low fat-free mass index
- Decreased muscle strength

In 2011, cachexia was redefined as: “a multifactorial syndrome defined by an ongoing loss of skeletal muscle mass (with or without loss of fat mass) that can be partially but not entirely reversed by conventional nutritional support” [3,7]. The revised diagnostic criteria for cachexia were the following:

- Weight loss > 5% over the past 6 months (in the absence of simple starvation)
- BMI < 20 kg/m^2^ and any degree of weight loss > 2%
- Appendicular skeletal muscle index consistent with sarcopenia and any degree of weight loss > 2% [3,7]

In 2019, Cederholm et al. published an updated definition of cancer cachexia, based on a consensus report from the global clinical nutrition community [8]. Cachexia was defined as chronic disease-related malnutrition with associated inflammatory changes. The diagnostic criteria involved one etiologic criterion from reduced food intake/assimilation or inflammation/disease burden and one phenotypic criterion from involuntary weight loss, low BMI, or low muscle mass [8]. According to Rier et al., cachexia involves a wasting process of both muscle and fat mass, unintentional weight loss, and systemic inflammation, in the presence of a malignant process or chronic disease [9].

Despite the above-mentioned variations in the definition of cachexia, its clinical course follows three main stages:

1. Pre-cachexia—the first stage of the disease, characterized by minor weight loss, anorexia, and glucose intolerance
2. Cachexia—the second stage of the disease progression, involving an unintentional weight loss > 5% within the last 6 months, sarcopenia or an unintentional weight loss > 2% in patients with BMI < 20%
3. Refractory cachexia—the last stage of the disease which has a decreased reversibility and is associated with a life expectancy below 3 months [5].

The clinical aspects of cancer-related cachexia are very complex. They include anorexia, inflammatory changes, weight loss in the adult population, growth delay in children, muscle degradation, and insulin resistance. Cachexia should be differentiated from starvation, malabsorption, hyperthyroidism, and age-related muscle mass loss [10].

## 3. Incidence and Epidemiology of Cancer-Related Cachexia

Cancer-related cachexia, the main cause of death in 22–30% of cancer patients, is still a poorly diagnosed condition [1]. It affects up to 74% of cancer patients, being directly responsible for 20–30% of cancer deaths [5].

The incidence of cachexia varies according to the type of malignancy, from 60–70% in gastroesophageal and pancreatic cancers, 40–50% in lung, colorectal and hematologic cancer to 20% in breast and prostate cancer, being highest in head, neck, gastric, hepatic and pancreatic cancers, according to studies published by Peixoto da Fonseca et al. and Han et al. [5,11]. Another study published by Jafri et al. states that the cachexia incidence depends on the tumor type, being the highest in pancreatic and gastric cancers (80–90%), the lowest in breast cancer and sarcoma, and present in 60% of lung cancer cases [2].

There are differences in gender distribution, with males being more affected than females. Cancer-related cachexia in patients over 60 years old occurs more frequently in males (40–60%) than in females (40–50%) [1]. According to Lim et al., males experience severe muscle loss more often than women (61% versus 31%) [1]. Both internal (hormonal levels and molecular differences) and external (delayed diagnosis, diet, smoking, alcohol consumption, lifestyle) factors can explain these differences between genders [1].

At the time of cancer diagnosis, malnutrition is present in 15–40% of cases and it increases after the start of treatment. It is associated with higher rates of treatment-related toxicity, poorer quality of life and physical functionality, and decreased overall survival rate [4,5].

## 4. Cachexia Versus Sarcopenia—What Do We Need to Know?

Muscle mass loss is seen in different catabolic conditions which share the common feature of muscle wasting. Although there are similarities between sarcopenia and cachexia and sometimes overlap, they are two different clinical entities and should be clearly distinguished [12,13].

The term sarcopenia comes from the Greek derivation: “Sarko” meaning “flesh” and “penia” meaning “poverty”, or “loss”. Therefore, sarcopenia means loss of muscle [12].

Sarcopenia is defined as a geriatric syndrome in which there is low muscle mass combined with low muscle strength or decreased physical performance. Cachexia, on the other hand, is a more severe entity in which there is wasting of both muscle and fat mass, with associated weight loss and systemic inflammation, in the presence of a malignant process or a chronic disease. Cachexia is a complex combination of both fat and muscle loss, hyperlipidemia, hyperglycemia, and inflammatory disturbances [9]. Most of the cachectic individuals have sarcopenia, but not all sarcopenic patients are cachectic [14].

Sarcopenic modifications which occur in a patient with a cancer diagnosis are part of the cancer cachexia syndrome. Systemic inflammation stimulates catabolic processes, anorexia and decreases nutrient intake, thus promoting skeletal muscle and adipose tissue loss. Early detection of sarcopenic patients and nutritional interventions are essential [15].

Sarcopenia is highly prevalent in cancer patients, due to physical inactivity, increased inflammation, hormonal and metabolic imbalances, and increased protein consumption. It is associated with higher rates of chemotherapy-induced toxicity, postoperative complications, and poor survival rate [16]. It is also very common among patients with dementia, diabetes, and respiratory diseases and is considered a modifiable condition and prognostic factor in cancer [17,18].

As diagnostic criteria for sarcopenia vary according to the consensus group cited, in the following lines we will emphasize three of the most frequently used ones. Sarcopenia was defined by the ESPEN Special Interest Groups (SIG) as a gait speed that was less than 0.8 m/s in the 4 m walking test or as the percentage of muscle mass that was less than two standard deviations (SDs) of the assessed mean of young people of the same age [19,20]. In addition, sarcopenia was defined by the European Working Group on Sarcopenia in Older People (EWGSOP) as grip strength < 20 kg for women and <30 kg for men, or gait speed < 0.8 m/s, and appendicular lean mass (ALM)/m^2^ below 5.67 kg/m^2^ for women and 7.23 kg/m^2^ for men [21]. The criteria for defining sarcopenia by the Society for Sarcopenia, Cachexia and Wasting Disorders (SCWD) was appendicular lean mass (ALM/h^2^) > 2 SDs below the mean of healthy individuals within the same ethnic group who are between the ages of 20 and 30; alternatively, it could be gait speed of ≤1.0 m/s or walking distance of <400 m over a 6 min walk [20,22].

Appendicular skeletal muscle mass measured by dual-energy X-ray absorptiometry (DXA) below 2 SDs than the mean of a healthy young adults group same as CT measured skeletal muscle index (SMI) at the level of third lumbar vertebra below the cut-off values are also diagnosing for sarcopenia [14].

Sarcopenia is considered primary when it is age-related, and secondary when it is caused by poor nutrition, malabsorption, endocrine and neuro-degenerative disorders, reduced physical activity, and cancer cachexia [14].

Sarcopenia prevalence is approximately 10% in females and males and is expected to increase dramatically worldwide, from 50 million in 2010 to 200 million in 2050 [23]. It is influenced by social factors, being higher in elderly people who live in residential care (14–33%) as compared to elderly living in the community (1–29%) [24]. Sarcopenic patients are at higher risk of fractures, falls, physical impairment, and mortality [12].

Regarding cachexia, according to a study published by Tagliafico et al., it can be considered a type of secondary sarcopenia caused by malnutrition and weight loss in severe chronic illnesses such as heart failure, kidney disease, and cancer [12].

The prevalence of cancer-related cachexia varies between 13 and 61% depending on tumor location. Sarcopenia prevalence varies between 38 and 70%, being highest in lung cancer patients (ranges from 47% to 79%, with a median of 56%) and in pancreatic cancer patients (ranges from 44% to 89%, with a median of 56%). A study conducted by Ryan et al. revealed that among cancer patients from Ireland and the UK, 34% had at least 5% weight loss annually and 35% of patients had sarcopenia [25].

Quantitative or qualitative analysis of muscle mass is not enough to differentiate sarcopenia from cachexia. However, some clinical features are more characteristic of cachexia rather than sarcopenia, such as rapid weight loss with failure of nutritional support and altered biochemistry [9]. The main differences encountered between cachexia and sarcopenia can be found in Table 1. 

## 5. Pathophysiological Mechanism of Cancer Cachexia

The exact mechanism by which cancer cachexia leads to muscle atrophy is not completely understood. Numerous studies have focused on a better understanding of this complex metabolic process, aiming to improve patients’ quality of life and overall survival. Decreased skeletal muscle protein synthesis and increased degradation processes take place during cancer cachexia. Molecules like pro-inflammatory cytokines, transcription factors, insulin-like growth factor 1 (IGF-1), kinases, proteins, and abnormal expression of angiotensin II (Ang II) are involved in the complex process of cancer cachexia [26].

The three main mechanisms involved in the pathophysiological mechanism of cancer cachexia are metabolic dysregulation and negative energy balance, which lead to weight loss, increased proteolysis and lipolysis, and neurohormonal imbalances [27].

**Figure 1:**
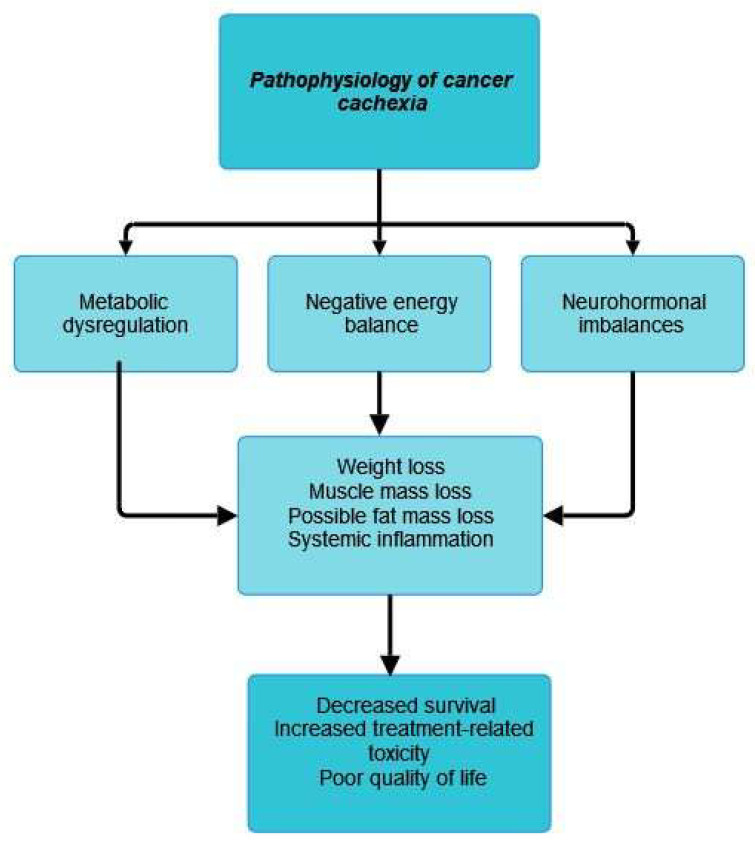
Pathophysiology of cancer cachexia.

In the following lines, we emphasize some of the most important mechanisms involved in cancer cachexia development. In general terms, the pathophysiological feature of cancer cachexia is an imbalance in protein and energy that is brought on by several variables, including decreased food intake and disorders related to metabolism [26]. The process of skeletal muscle wasting in cancer cachexia has also attracted a lot of attention due to autophagy [27].The main pathophysiological mechanism involved in the development of cancer cachexia can be found in Figure 1. 

### 5.1. Inflammation, Cytokines, and Cancer-Related Cachexia

The interaction between the tumor and the body’s tissues leads to a systemic inflammatory response. Systemic inflammation is associated with negative nitrogen balance, increased energy consumption, and weight loss [28]. A systemic inflammatory response leads to a progressive impairment of the functional and nutritional status of the patient, a poor prognosis, and decreased survival of cachectic cancer patients [29]. The activation of the humoral immune system and the secretion of tumor cell-derived cytokines, such as tumor necrosis factor-alpha (TNF alpha), interferon-γ (IFN-γ), interleukin-1β (IL-1β), and interleukin-6 (IL-6) are part of the pathophysiology of cancer-related cachexia. Immune cells such as neutrophils, macrophages, T-cells, and bone marrow-derived suppressor cells are also involved in cancer cachexia pathogenesis [1,3].

The role of inflammation in cancer-related cachexia has been discussed in numerous studies and the role of cytokines in cancer cachexia progression is unequivocable. According to a study published by Donohoe et al., TNF-alpha and Proteolysis-inducing factor (PIF) are the main two substances involved in skeletal muscle atrophy of cachectic patients, by promoting protein degradation and decreasing protein synthesis [30]. TNF alpha, also known as cachectin, induces activation of the nuclear factor kappa-light-chain-enhancer of activated B cells (NF-kB). NF-kB is associated with a decreased transformation of protein into amino acids, leading to muscle atrophy [11]. Interleukin 6 (IL-6) leads to skeletal muscle wasting by promoting proteasome and autophagy protein degradation. It also affects adipose tissue, the gut, and the liver. Interleukin- 1 β (Il-1 β) induces neuroinflammation, proteolysis, lipolysis, decreased appetite, and increased resting energy expenditure. Recently, other cytokines have been described as being involved in the pathophysiological mechanism of cancer cachexia: TNF receptor (TNFR)-associated factor 6 (TRAF6), TNF-like inducer of apoptosis (TWEAK), leukemia inhibitory factor (LIF), and interferon-gamma (IFN-γ) [31,32,33,34].

Genetic variation in immunity might be related to some patients’ predisposition to developing cancer cachexia [35].

T-cells’ involvement in the cachectic syndrome is still understudied. Recent publications associated the total number of T-cells, CD3-CD4 cells, and phagocytes/granulocytes with the muscle mass status. A study published by Baazim et al. revealed the relationship between CD8-T cells and skeletal muscle wasting in mice [36]. In addition, another study published by Narsale et al. in 2019 evidenced a strong correlation between T-cells, body mass, muscle strength, and performance in a small cohort of patients with gastrointestinal cancer [37].

Growth differentiation factor-15 (GDF-15) has an important role in cachectic patients, as high levels of GDF-15 in the early stages of cachexia represent a poor prognostic factor for patients who experienced weight loss and its overexpression stimulates muscle atrophy. Growth differentiation factor-11 (GDF-11), another member of the transforming growth factor -β (TGF-β) family, acts as an inhibitor of skeletal muscle growth, and its concentration is directly related to the degree of cardiac and skeletal muscle atrophy [10].

According to Donohoe et al., rodent tumor models that had increased levels of pro-inflammatory cytokines also experienced increased weight loss. Parathyroid hormone-related peptide (PTHrP), a tumor-derived circulating factor leads to decreased levels of both transferrin and albumin [30]. There is still a debate if the production of cytokines in cachectic patients is due to the tumor itself or the response of host inflammatory cells to tumor cells [30].

### 5.2. Lipolysis, Proteolysis, and Cachexia

Cancer cachexia leads to weight loss, due to both muscle and adipose tissue loss [10].

Increased muscle protein catabolism, promoted mainly by ATP ubiquitin-dependent proteolytic pathway and to a lesser extent by other proteolytic pathways, such as calcium/calpain pathway and lysosomal cathepsins B, H, F, and L, is involved in cancer cachexia [10]. Two major atrophy-related genes: atrogin-1 (also known as MAFbx) and MuRF1 (muscle RING finger protein 1)) are also cited [1].

Cancer-induced cytokines formation leads to the reduction of myoblast determination protein 1 (MyoD), a transcription factor involved in muscle development, leading to muscle wasting [10]. Adipose tissue and skeletal muscle wasting are strongly interrelated, as functional lipolysis in-duces muscle wasting by promoting excessive oxidation of free fatty acids in skeletal muscle and release of adipokines, among which leptin seems to be the most important in the context of cancer cachexia [35].

The role of proteolysis-inducing factor (PIF) in cancer-induced cachexia was postulated by Tournadre et al. in their study. It is related to skeletal muscle catabolism in experimental models and a decrease in total muscle mass by decreasing protein synthesis and increasing protein degradation. PIF also activates the ATP-dependent ubiquitin-proteasome pathway (UPP system), the most important system involved in myofibrillar protein degradation [38]. Proteolysis is also stimulated by leptin, a hormone produced by enterocytes and adipocytes and secreted in small amounts in cachectic patients, which promotes decreased appetite and increased insulin resistance, leading to additional muscle proteolysis [9,35].

The role of lipolysis in the pathophysiology of cancer cachexia was also described by Tournadre et al. in their study. Increased catabolic processes lead to a complete loss of white adipose tissue (WAT) and a reduction of muscle mass [38]. LMF (lipid mobilizing factor), a tumor-produced substance is also involved in the pathophysiology of cachexia-related lipolysis. It stimulates the production of cyclic adenosine monophosphate (AMP) with the outcome of sensitizing adipose tissue to lipolytic stimuli, thus stimulating lipolysis and promoting adipose tissue wasting [10]. The tumor factor zinc-alpha 2 glycoprotein also leads to loss of adipose tissue mass [10].

Another key factor in the pathophysiology of cancer cachexia is myostatin. It binds to the activating type II B receptor, leading to decreased muscle differentiation and growth. IGF-1 (insulin-like growth factor 1) is sensitive to food intake and blocks in physiological conditions the myostatin pathway, thus preventing cachexia. In cachectic patients, myostatin is overexpressed and IGF-1 inhibited [3,39]. Substances like leptin, ghrelin, and growth differentiation factor-15 (GDF-15) also regulate the appetite center [10].

The role of muscle regeneration in cancer cachexia is still discussed. Cancer-related muscle wasting is partially due to low muscle regeneration in response to muscle wasting processes dependently or independently of protein synthesis or cellular signaling. A study conducted by Lim et al. identified decreased levels of cell cycle and myogenic-associated markers of muscle atrophy, like Myogenin, myoblast determination protein 1 (MyoD), paired box protein 7(Pax7), and Cyclin D1 in tumor-bearing mice before the onset of weight loss, suggesting that muscle atrophy caused by decreased muscle regeneration might be initiated at the onset of neoplastic processes [1].

New studies draw attention to neoepitopes (proteins produced by post-translational modifications of existing molecules) as new potential biomarkers of muscle wasting. Extracellular matrix proteins (laminins) and sarcomeric proteins (tropomyosin, myosin, actin, and troponin) are included here. In addition, the N-terminal propeptide of type III procollagen (P3NP) is associated with alterations in lean body mass and appendicular skeletal muscle mass. It is also a predictor of anabolic response to testosterone and growth hormones [40].

**Figure 2:**
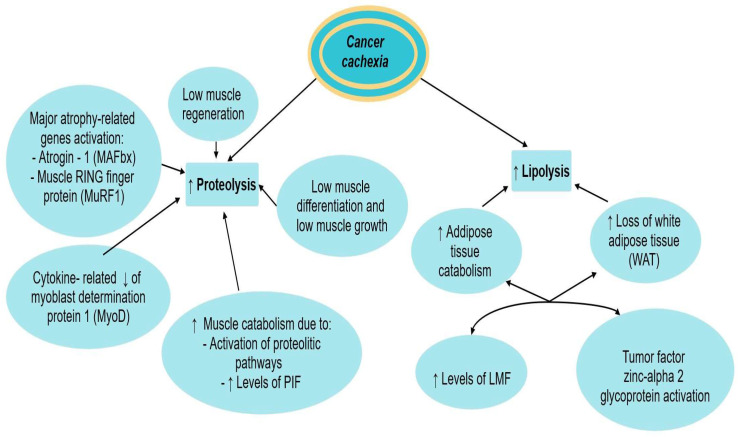
Proteolysis and lipolysis in cancer cachexia. ↑ equals increased, ↓ equals decreased.

The involvement of both lipolysis and proteolysis in the development of cancer cachexia has been described in Figure 2. 

### 5.3. Mitochondrial Dysfunctions and Cancer Cachexia

Mitochondrial changes during cancer cachexia are still understudied. According to Lim et al., mitochondrial oxidative stress processes and reactive oxygen species (ROS) generation precede the development of cancer cachexia in tumor-bearing mice. There is impaired mitochondrial dynamics, autophagy, quality control, and reduced content of mitochondrial fusion regulatory proteins [1]. The role of mitochondria in cancer cachexia pathogenesis was also discussed by Tournadre et al. Mitochondrial alterations lead to poor mitochondrial quality and activity, malfunction of mitochondrial metabolism and oxidative phosphorylation processes, DNA damage, and altered mitochondrial dynamics [38].

Many studies relate mitochondrial dysfunction to sarcopenic alterations. Recent studies promote the importance of growth differentiation factor 11 (GDF11) in age-related skeletal muscle function. Sinha et al. demonstrated in their study that GDF11 administration to aged mice improved myofibrillar and mitochondrial morphology, promoted the formation of the neuromuscular junction, and improved muscle regeneration and endurance [41]. However, these results were not approved by Egerman et al. who demonstrated in their study that GDF11 administration inhibits not stimulates muscle regeneration [42].

The role of mitochondrial dysfunction in cancer cachexia has also been postulated by Tournadre et al. Mitochondrial DNA mutations, altered mitochondrial antioxidant properties, and accumulation of reactive oxygen species (ROS) lead to the alteration of motor neurons and myofibrils, stimulating muscle wasting phenomena of cachectic patients [38].

**Figure 3:**
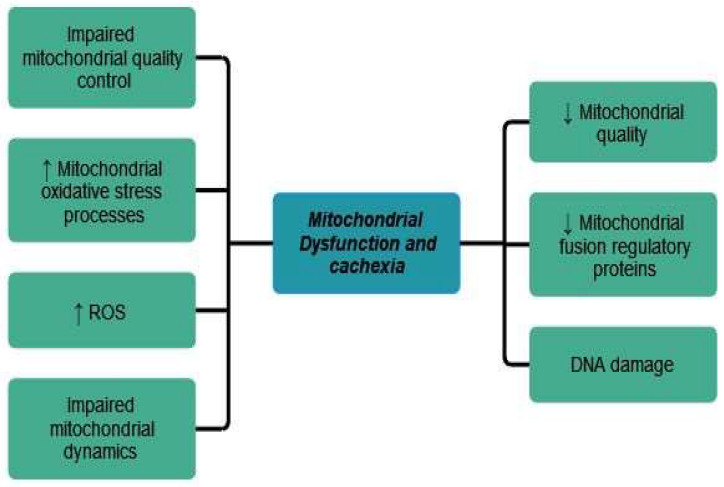
Mitochondrial dysfunction in cancer cachexia. ↑ equals increased, ↓ equals decreased.

The main mitochondrial dysfunction and its relationship to cancer cachexia has been shown in Figure 3. 

### 5.4. Gut Microbiota and Cachexia

Gut microbiota changes are related to muscle atrophy by affecting skeletal muscle homeostasis. Gut microbiota produces metabolites like indoxyl sulfate which is thought to be a cause of muscle atrophy and urolithin A (UA) which is related to increased mitophagy in skeletal muscle [17,43].

### 5.5. Appetite Center and Cachexia

The pathophysiology of appetite regulation in cancer patients is still under debate. Two sets of neurons located in the arcuate nucleus of the hypothalamus are involved: the neuropeptide Y (directly stimulates appetite or by releasing other orexigenic proteins) and the melanocortin system (leads to decreased food-seeking behavior and lean body mass and increased basal metabolic rate) [30].

In cancer patients, the production of inflammatory cytokines dysregulates the balance of the appetite center. Substances like leptin, ghrelin, and growth differentiation factor-15 (GDF-15) are also involved [10]. Cancer alters zinc homeostasis as a consequence of the acute phase response to inflammatory cytokine activity. Low zinc levels are associated with hypogeusia, thus further contributing to low food appetite and anorexia in cancer patients [38]. Lactate is also involved in the pathophysiological mechanism of cancer cachexia. Increased levels of lactate are found in cachectic patients, lactate being an anorexic agent. In addition, cachectic patients also experience high plasmatic levels of tryptophan, the precursor of serotonin. Serotonin negatively influences food intake by activating anorexigenic neurons like pro-opiomelanocortin (POMC) and cocaine- and amphetamine-regulated transcript (CART) in the melanocortin system [44].

**Figure 4:**
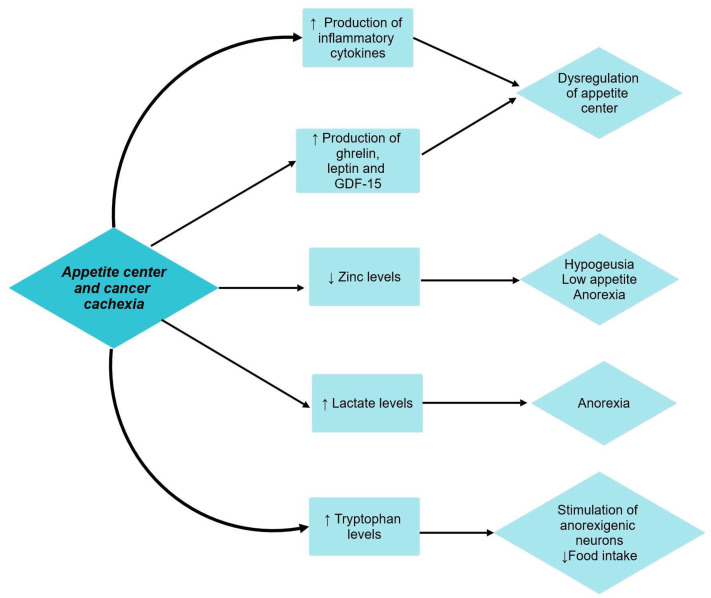
Appetite center and cancer cachexia. ↑ equals increased, ↓ equals decreased.

The relationship between cancer cachexia and its effect on the appetite center has been shown in Figure 4. 

## 6. Clinical Impact of Cancer Cachexia

Cancer cachexia has a complex clinical impact and dramatically affects patients’ quality of life [1]. Multiple molecules are involved in cancer-related cachexia, varying from cytokines, neuro-transmitters, tumor-derived factors, and neuropeptides. High levels of C-reactive protein (CRP), interleukin 6 (IL- 6), and interleukin 10 (IL-10) are associated with poor performance, weight loss, and worse prognosis in cancer patients [38].

### 6.1. General Consideration

Cancer cachexia leads to overall weakness, fatigue, decreased physical performance, and quality of life [30]. It affects up to 80% of cancer patients and is directly responsible for at least 20% of cancer deaths [40]. Moderate to severe weight loss, depending on cancer type, was diagnosed in 30–70% of cancer patients, according to a study based on more than 3000 patients. The most pronounced weight loss was seen in patients diagnosed with pancreatic, lung, head, gastric, and colorectal cancers, while breast and hematological cancers were associated with a lower risk. According to a study published by Berardi et al., patients diagnosed with solid tumors are more likely to lose at least 10% of their initial weight [40]. The degree of weight loss which is associated with a poor prognosis has not been exactly established. A longitudinal study showed that a weight loss of 2.5 kg during a period of 6–8 weeks is enough to negatively influence the performance status of the patient. Death is associated with a weight loss of 30% [30].

Unintentional weight loss, decreased quality of life, performance status, increased pro-inflammatory cytokines, and fatigue are all linked to cancer cachexia [40]. Inflammatory cytokines are produced either by the tumor itself, the immune system, or the muscle or adipose tissue [40]. Reduced food intake, early satiety, and abnormal eating behavior should always be evaluated in cancer patients [40].

Cancer cachexia is associated with alterations in hemoglobin, albumin, and C-Reactive Protein (CRP) levels [30].

Cachexia score (CASCO) was developed by Argilés et al. to quantitatively evaluate cachectic cancer patients. It takes into consideration inflammatory changes/immunosuppression/metabolic disturbances, body composition and weight loss, anorexia, and quality of life (QOL) [45]. This score ranges from 0 and 100 and classifies cachexia into mild (<25), moderate (26–50), severe (51–75), and terminal phase (76–100) [3].

**Figure 5:**
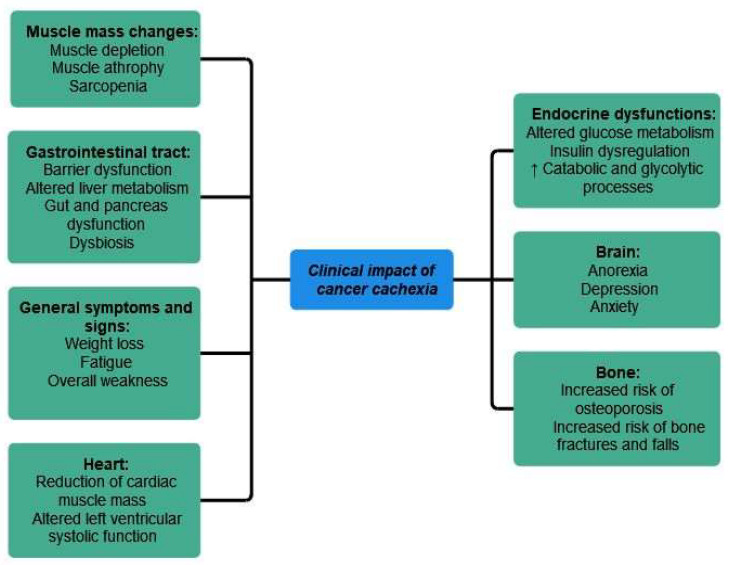
Clinical impact of cancer cachexia. ↑ equals increased.

The main clinical impact of cancer cachexia has been summarized in Figure 5. 

### 6.2. Muscle Mass and Cachexia

Muscle homeostasis is affected in cachectic patients, leading to reduced muscle function, mass, and strength [40]. Muscle depletion is the most important clinical feature of cancer cachexia [25]. Cancer-related muscle atrophy has been extensively studied, however there is still no concrete understanding of it. Muscle wasting is linked to physical impairment, reduced tolerance to treatments, and decreased overall survival rates [46]. In addition, the amount of muscle mass that a patient has is involved in his response to immunological therapies [47].

A decreased amount of skeletal muscle mass in cancer patients is associated with a poorer quality of life, increased fatigue, and a negative prognosis. A meta-analysis of adult patients with solid tumors demonstrated the negative prognosis of patients with low skeletal muscle index (SMI) [48].

Sarcopenia leads to an increased length of hospitalization days, and post-operative infections, especially in patients over 65 years old. A study published by Prado et al. demonstrated that sarcopenic obesity was linked to poorer functional status and overall survival rates in respiratory and gastrointestinal cancers [14,49]. The degree of physical activity impairment in cachectic patients is substantial, leading to decreased performance status, social life, and daily activity limitations [30].

Further studies are needed to achieve a better understanding of how body composition influences cancer patients’ outcomes [48].

### 6.3. Gastrointestinal Tract

The effects of cancer cachexia on the gastrointestinal tract are multiple and complex. According to Delzenne et al., the gastrointestinal tract alterations during cancer cachexia vary from barrier dysfunction, dysbiosis, and digestive impairment, leading to metabolic alterations and systemic inflammation [35,50]. The pancreas, liver, and gut are all affected by cancer cachexia [35].

Liver metabolism is altered during cancer cachexia, with mitochondrial dysfunction being directly linked to cachectic processes. According to Dumas et al., increased fatty acid accumulation in hepatic mitochondria, the same as decreased oxidative phosphorylation and increased production of oxygen reactive species (ROS) were observed in rats with cancer cachexia [51]. Cachexia is associated with increased liver mass and liver fibrosis, caused by increased collagen storage and elevated matrix metalloproteinase (MMPs) [1]. Studies suggest that cachectic patients with stage 3 and 4 pancreatic cancer have more numerous liver infiltrating macrophages and a poorer nutritional status. According to Thibaut et al., cachectic mice suffer from derangements in hepatobiliary secretion due to “inflammation-induced cholestasis” and in bile acid metabolism [35,52].

Cancer cachexia also affects adipose tissue composition and distribution. It contributes to the loss of WAT (white adipose tissue) by increasing lipolysis, which precedes the loss of muscle mass [5]. Recent studies found a possible association between low muscle mass and decreased gut microbiota diversity. Intestinal microbiota diversity seems to be directly influenced by general performance status and physical activity. Patients diagnosed with anorexia nervosa and patients who are sedentary and aging have a less diversified gut microbiota composition and decreased muscle function. The gut is also involved in muscle mass loss in cachectic cancer patients due to barrier dysfunction, nutrient malabsorption, and chronic systemic inflammation [40]. Numerous studies suggest that cachexia is linked to gut dysfunction, but there is no clear evidence of exactly which phase of cancer cachexia gut dysfunction occurs [35]. However, according to Ferrara et al., alterations in the gut appear at the onset of cachexia [35].

### 6.4. Endocrine and Metabolic Dysfunctions

Cancer cachexia is associated with imbalances in glucose metabolism, with insulin resistance being frequently associated [35]. Insulin resistance in cancer patients is different from the one in type-2 diabetes mellitus, it is characterized by normal fasting glucose levels associated with any insulin level [35,53]. This phenomenon can be explained by the use of glucose by tumoral cells to promote cancer growth [11].

Insulin dysregulation is a common host adaptation to cancer growth, but cachexia significantly interests only a variety of cancer types, according to a study published by Dev et al. in 2018 [53]. The concept of insulin resistance and insulin sensitivity in cancer patients is still controversial, as insulin resistance (IR) was described in some types of cancer [54,55] and in early stages of cachexia in mice models, whereas other studies showed no correlation between cancer patients, cachexia, and IR [56].

There are also differences in insulin sensitivity between cancer patients with or without associated cachexia, due to hormonal imbalances (vitamin D, testosterone, GLP-1, glucagon, ghrelin, apelin) and adipose tissue modifications. Cancer cachexia also impacts pancreatic exocrine function and digestive enzyme secretion. Their dysregulation directly influences nutrient absorption, gut microbiome, and function and is directly linked to adipose tissue wasting, thus promoting weight loss in cancer patients [35].

Catabolic and glycolytic processes are upregulated in cachectic patients, leading to increased plasmatic levels of amino acids and lactate. These products will further stimulate gluconeogenesis processes, causing increased glucose levels, needed for tumor growth, according to a study published by Lim et al. [1].

### 6.5. Heart

The cardiac muscle is also affected by cancer cachexia. A reduction of cardiac muscle mass and altered left ventricular systolic function are seen in cachectic patients. Cancer patients diagnosed with cachexia frequently suffer from shortness of breath, decreased exercise tolerance, and fatigue [1]. According to Ferrara et al., muscle wasting affects both heart and diaphragm proteins, leading in severe cases to cardiac arrest and respiratory failure [35].

Recent studies showed that in preclinical models, heart weight loss is frequently present when cachexia is established. The underlying pathophysiological mechanism is linked to increased heart autophagy and ubiquitin-proteasome pathways, leading to increased oxygen and energy consumption and negative energy balance [35,57,58].

According to Lim et al., cardiac atrophy and cardiac dysfunction are correlated with the degree of skeletal muscle atrophy [1]. Still, cardiac cachexia remains understudied; however, there is evidence that cardiac dysfunction associated with cancer cachexia is directly linked to cancer death [1,5].

### 6.6. Bone

Recent studies suggest that the relationship between skeletal muscles and bones is more complex than purely mechanical and structural. Myokines such as IL-6, irisin, and myostatin are involved in bone formation and resorption processes, according to a study published by Berardi et al. Other substances involved in muscle mass homeostasis are osteocalcin (positive effect on muscle mass and function), transforming growth factor (TGF), and Wingless-type 3 protein (Wnt-3) (involved in myocyte differentiation and, respectively decrease muscle function via oxidative stress) [40]. Sarcopenia, a process directly linked to cachexia, is associated with an increased risk of osteoporosis. This is due to the hyperexpression of myostatin, a myokine involved in muscle mass, bone mass, and osteoclast formation. The relationship between osteoporosis and sarcopenia is more complex, involving vitamin D levels, same as growth, sex, and insulin-like growth hormones. Sarcopenia also increased the risk of bone fractures and falls and is associated with increased morbidity and mortality [48].

### 6.7. Brain

An active lifestyle positively impacts memory, sleep, hippocampal neuroplasticity, neurogenesis, and vascularization. It also decreases the risk of depression and anxiety. Brain-derived neurotrophic factor (BDNF) is associated with the majority of these positive effects. BDNF is produced and released during exercise, but there are other myokines and metabolites released by skeletal muscle that are also involved in BDNF activation [40].

Cancer cachexia is associated with systemic inflammation. Systemic inflammation affects the hypothalamus homeostasis, activating anorexigenic neurons ((proopiomelanocortin (POMC) and cocaine-and-amphetamine regulated transcript (CART)) and inhibiting orexigenic neurons (neuropeptide Y (NPY) and the agouti-related protein (AgRP)), according to Argilés et al. [35,59].

Cachexia also affects patients’ well-being, as depression and anxiety are more frequent in cancer-related cachexia [35,60].

A brief overview of the main clinical impact of cancer cachexia, in regards to the structural and functional changes encountered and their main consequences has been provided in Table 2. 

## 7. Cachexia in Different Types of Malignancies

Cachexia is associated with different types of malignancies and is more common in advanced-stage malignancies. Tumor characteristics and inflammatory changes are involved in its pathogenesis [1]. According to a study conducted by Arthur et al., between 15 and 50% of patients with cancer experience cachexia, the highest incidence being in the lung, stomach, pancreas, esophageal, and Kaposi’s sarcoma malignancies [61]. The study also demonstrated that males are more affected than females of all cancer types, the biggest gender difference being attributed to Kaposi sarcoma [4,61]. A systematic review of 21 studies revealed that cachexia prevalence in patients at risk of developing it was approximately 30% in Europe and the U.S.A. The highest incidence was found in patients diagnosed with cancer of the liver (50%), pancreas (45.6%), head and neck (42.3%) [4]. Pancreatic and lung cancer tumors have specific gene expression profiles of cachexia-inducing factors that predispose patients to develop a wasting syndrome. Malnutrition appears more frequently in gastrointestinal malignancies, like esophageal and gastric cancers [4].

According to Bossi et al., malnutrition is the highest among gastroesophageal, pancreas, neck, and head tumors [4]. In advanced esophageal cancer, its prevalence increased from 16% at the time of diagnosis to 35% a year after diagnosis, with older patients being slightly more affected (44.6%) [4]. The same results were confirmed by Bozzetti et al. and Planas et al., who using the Nutritional Risk Screening reported an increased nutritional risk of up to 62.5% for esophageal and 66.7% for pancreatic cancers [62,63]. Different risk screening tools demonstrated even higher rates (70.6% for gastroesophageal and 70.6% for pancreatic tumors) [4]. A study published by Ruan et al. confirmed that gastric and pancreatic cancer patients experience the highest rate of weight loss, up to 30% of their pre-morbid body weight, with older people being more affected [15]. When using BMI values and percentage weight loss over time, Pressoir et al. revealed a malnutrition prevalence of 49.5% for upper digestive tumors [64]. These results were confirmed by another Italian study conducted on 1951 patients, which demonstrated a malnutrition prevalence of 40.2% using the mini nutritional assessment system [4]. Major loss of function was associated with cachexia in 41% of lung and stomach cancer patients, 40% in pancreatic and 37% in esophageal cancer patients, while hematologic malignancies have malnutrition rates of 34–36.8%, according to Bossi et al. [4].

Dermatological malignancies and associated sarcopenic alterations were studied by Chu et al. and Takenaka et al. On one hand, a study conducted by Chu et al. investigated the association between CT-measured low skeletal muscle density (SMD) in patients diagnosed with metastatic melanoma and treated with ipilimumab and their outcome. The results showed that low SMD was associated with poorer progression-free survival (PFS) and overall survival (OS) compared with high SMD. In addition, the study revealed that patients with low SMD had an elevated neutrophil-to-lymphocyte ratio (NLR) more frequently than high SMD patients, thus suggesting inflammation [65]. On the other hand, Takenaka et al. investigated if sarcopenia can be used as a predictive factor for cancer patients treated with an immune checkpoint inhibitor (ICI), melanoma and NSCLC being the most investigated tumors. Immune checkpoint inhibitor (ICI) therapy is the most frequently used type of immunotherapy in cancer patients. The study revealed that cancer patients with sarcopenia had a worse prognosis, disease control rate, and progression-free survival [66].

Lung cancer-associated malnutrition ranges from 20.9% to 45.3%. Lung cancer-associated sarcopenia was estimated to be 52.8% [4]. A study published by Arthur et al. in 2016 revealed that lung cancer patients admitted in the study had in a percentage of 5.29% associated diagnosis of cachexia [61].

Increased risk of malnutrition has been linked to genitourinary malignancies in the case of 28.6% of patients diagnosed with prostate/testicle neoplasms, 33.3% of patients diagnosed with kidney/bladder cancers, and 44.8% of patients diagnosed with bladder/uterus tumors. Generally speaking, patients with colorectal and breast cancers have a lower prevalence of malnutrition [4].

There is an increased need for developing predictive tools to identify cachexia in its early stages, decrease its severity, and improve patients’ outcomes, especially in high-risk malignancies [1].

## 8. CT Imaging and Its Role in Cancer Cachexia

Cancer cachexia, a multi-organ syndrome with complex metabolic and endocrine involvement, has a negative impact on body composition and tissue quality. Novel non-invasive techniques are needed to properly assess longitudinal body composition changes. Medical imaging has the greatest potential to properly assess these changes and improve cachectic patient outcomes [5].

Imaging techniques offer a detailed evaluation of a patient skeletal muscle mass and adipose tissue. Computed tomography (CT) and Magnetic Resonance Imaging (MRI) are used to properly evaluate body composition, but their use is influenced by limited availability, high cost, and ionizing radiation exposure in case of CT evaluation [67].

CT is the current gold standard for the proper analysis of sarcopenic, cachectic, and frail patients, as it can evaluate body composition and distinguish between different tissues based on the specific attenuation of each tissue measured in Hounsfield units (HU).

Skeletal muscle index (SMI—cm/m^2^), skeletal muscle area (SMA—cm^2^), and muscle radiation attenuation (MRA—HU) are the most often used metrics that measure muscle mass [23]. An additional parameter that can be ascertained with CT images at the level of L3 is the total abdominal muscle area (TAMA). TAMA is highly correlated with total body muscle mass and can be corrected to the patient’s height, resulting in a skeletal muscle index (cm^2^/m^2^). Muscle mass analysis can be performed quickly, limiting the dose of ionizing radiation [9].

The above-mentioned parameters are obtained by analyzing a single CT slice, usually at the level of the L3 vertebral body, the most appropriate anatomical site for measuring skeletal muscle and adipose tissue [67,68]. The cross-sectional imaging at the level of the third lumbar vertebra (L3) highly correlates with total body muscle mass, fat-free mass, fat mass, and appendicular skeletal muscle mass and is the preferred method to assess skeletal muscle mass [67,68,69]. The L3 vertebra is visible in almost all CT scan protocols used for the diagnosis and monitoring of cancer patients: chest-abdomen (T1-L4), chest-abdomen-pelvis (T1-L5), and abdomen scans (T10-L4). It correlates best with true volumetric body metrics in non-malignant populations and according to studies published by Tolonen et al. and Baracos et al., L3 measurement was also validated for cancer patients [67,68]. This single-slice measure contains information about muscle tissue (erector spi-nae, quadratus lumborum, transversus abdominalis, external and internal oblique abdominals, and rectus abdominus), subcutaneous and visceral adipose tissue [67,68]. The skeletal muscle attenuation threshold on CT scans ranges from −29 to 150 HU [70]. The drawback of the L3 landmark is that it cannot be used for lung cancer patients who undergo limited chest CT scans (T1-L1). Several studies have thus proposed alternative chest CT landmarks, like L1 and L2 for skeletal muscle mass and index, but these landmarks have not been adequately validated in large cancer populations [71,72,73,74].

Besides the L3 level, the T4 level can be used to evaluate total muscle area [12]. Also, Derstine et al. reported muscle cut-offs for diagnosing sarcopenia from T10-L5, extending sarcopenia evaluation to both chest and pelvis CT scans and increasing the clinical application of this imaging technique [24,72]. CT is superior to DXA in body composition analysis, as it can properly distinguish between adipose tissue (subcutaneous and visceral fat) and skeletal muscle [14]. Adipose tissue can be evaluated either by directly identifying fatty areas inside the muscle or by evidence of decreased HU attenuation on CT images [12].

Parameters like total lean body mass, total fat mass, lean and fat body mass indices (normalized for height), subcutaneous fat-to-muscle ratio, and visceral-to-subcutaneous adipose tissue ratio can also be calculated using CT acquisitions [14]. The cut-off values for CT-determined sarcopenic alterations using SMI depend on gender, muscle group, BMI, and the anatomical level used for evaluation. Amini et al. conducted a meta-analysis that concluded that the most frequently used cut-off values for L3 calculated SMI ranges from 39 to 41 cm/m^2^ for women and 52 to 55 cm/m^2^ for men [67]. CT-derived SMI and SMD offer an objective and complex insight into the relationship created between the tumor and the host itself [75].

To achieve concluding results, non-contrast CT images must be used for a proper evaluation of skeletal muscle mass, as intravenous contrast media injection increases muscle attenuation and decreases results accuracy [24]. Software development is also important in the process of muscle mass analysis. The most frequently used software is ImageJ version 1.48 and Slice-O-Matic version 5.0, both of them sharing excellent intra-observer and inter-observer agreement [23,76]. The advantage of CT imaging for the diagnosis of low skeletal muscle mass is that it can be utilized both in prospective and retrospective studies, as it is routinely used for the proper initial evaluation and follow-up of cancer patients [24]. Low muscle mass is associated with increased length of hospitalization, decreased tolerance to treatment, worse outcomes, and higher mortality [12]. CT-determined loss of skeletal muscle mass has an important clinical significance in cancer patients, as it is directly correlated with decreased survival in head, neck, breast, prostate, gastrointestinal, and urinary tract malignancies. In addition, CT-determined loss of skeletal muscle mass at the level of L3 is correlated with decreased survival, increased hospitalization days, and higher 30-day mortality in ICU patients [67]. There is a frequent omission of cachexia-related deaths in national databases, raising awareness that cachexia still needs to be investigated to be better understood [5]. Radiology and radiologists could have a central role in the proper and early diagnosis of muscle and adipose tissue alterations. Sarcopenia is still an underdiagnosed condition and medical imaging could be the key to the early recognition and proper management of sarcopenic patients [12].

In addition, artificial intelligence progression allows the easy conversion of CT images into data that can be easily processed for quantitative analysis of muscle mass. This process is called radiomics and offers a three-dimensional approach to whole body tissue, transitioning from manual 2D segmentation images to automated 3D volumetric assessment. It improves the accuracy of body composition assessment and helps in providing a personalized treatment plan for the patient, with the final goal of improving overall survival rates [5].

## 9. Prevention and Therapeutic Options in Cancer Cachexia

In recent years, there has been an increased interest in finding proper prevention methods, management strategies, and therapeutic approaches for cancer cachexia [77].

Finding optimal cancer-cachexia treatment remains under debate, as cachexia is a systemic multiorgan syndrome with multiple associated metabolic and inflammatory components. There is a huge need for a multimodal approach of these patients, combining pharmacological therapies with physical exercise [77].

Drug treatment of cachectic patients follows a very complex approach, with multiple substances being cited as possible therapeutic strategies.

Corticosteroids showed good effects in terms of preserving QOL and body weight. However, their use is restricted to end-of-life care due to the high rate of side effects with prolonged use. The single use of non-steroidal anti-inflammatory drugs has not been demonstrated to enhance nutritional status or metabolism, but, if taken in conjunction with other treatment modalities, they may prevent cachexia development. As in the case of corticosteroids, unnecessary administration is advised to be avoided due to frequent adverse effects. Anti-cytokine therapy was also proposed as a therapeutic option for cancer cachexia patients, with beneficial effects on decreasing inflammation [78].

Gastrointestinal hypermotility drugs improve appetite loss, while anamorelin hydrochloride, a drug that promotes ghrelin-like actions and increases GH secretion and appetite was also cited as a possible therapeutic strategy [78].

Currently, the first nutritional care strategy advised for increasing oral nutritional intake is the use of oral supplements (ONS). In addition, eicosapentaenoic acid administration was related to an improved quality of life, decreased PIF production and muscle degradation [78]. L-carnitine, coenzyme Q10 (CoQ10), and branched-chain amino acids showed beneficial effects in terms of decreasing protein degradation and anorexia [78].

Exercise was also cited as a promising strategy to prevent cancer-related cachexia, as it was related to increased muscle function and strength, decreased fatigability, and improved quality of life. In addition, exercise induces anti-inflammatory effects, and improves muscle anabolism and function [77,79].

Time is also very important when treating cancer cachexia, as early diagnosis and therapeutic intervention are key factors for improving patients’ overall condition and outcome. This raises awareness of the importance of identifying specific biomarkers for the early identification of patients at high risk of developing cancer-related cachexia and thus, proper intervention and management of these high-risk patients [79].

## 10. Conclusions and Future Directions

Cancer remains a major public health problem, with cancer-related cachexia being an additional risk factor for increased mortality and decreased quality of life. Cachexia involves complex metabolic disturbances, altered homeostasis, increased systemic inflammatory response, muscle mass imbalances, and weight loss. As stated before, cachexia does not equal sarcopenia, although these two conditions may sometimes overlap. A better understanding of the complex pathophysiological implications of cancer-related cachexia is needed to improve cancer patients’ outcomes and prognosis.

In recent years, there has been enormous progress in better understanding the clinical implications of cachexia. Multiple cytokines, neurotransmitters, tumor-derived factors, and neuropeptides are involved in the pathophysiology of this condition, inflammatory changes being a key factor of cancer cachexia pathogenesis. Further studies are needed to develop efficacious biomarkers for the early detection of cachexia and precise treatment.

Studies performed on preclinical models are also of huge interest for a better understanding of cancer cachexia phenomena.

The role of imaging techniques (CT and MRI) in the detection of muscle wasting is unequivocal, with radiology playing a central role in cachexia and sarcopenia diagnosis. In addition, progress in artificial intelligence allow for a three-dimensional approach to the whole body tissue, thus improving the process of body composition assessment.

For patients with cancer-associated cachexia, a multidisciplinary approach is needed. Clinical, laboratory, and imaging assessment, as well as nutritional and psychological support are equally involved in the proper management of this pathological condition.

## Figures and Tables

**Table 1:** Main differences between cachexia and sarcopenia.

	Cachexia	Sarcopenia
DEFINITION	A severe entity defined by muscle and weight loss, with or without fat loss combined with systemic inflammation, in the presence of a malignant process or a chronic disease	Geriatric syndrome defined by low muscle mass, low muscle strength, or physical performance
DIAGNOSTIC CRITERIA	1. In 2008, cachexia was defined based on: ◦ unintentional weight loss of at least 5% in the last 12 months or less in the presence of an underlying disease and at least three of the following: fatigue, anorexia, abnormal biochemical lab results, low fat-free mass index, decreased muscle strength 2. In 2011 cachexia was defined based on: ◦ weight loss > 5% over the past 6 months (in the absence of simple starvation) BMI < 20 kg/m^2^ and any degree of weight loss > 2% appendicular skeletal muscle index consistent with sarcopenia and any degree of weight loss > 2% 3. In 2019, cachexia was defined based on a consensus report from the global clinical nutrition community by meeting: ◦ one etiologic criterion from reduced food intake/assimilation or inflammation/disease burden one phenotypic criterion from involuntary weight loss, low BMI, or low muscle mass	1. ESPEN Special Interest Groups (SIG) definition criteria: ◦ the percentage of muscle mass ≥ 2 standard deviations (SDs) below the measured mean of young adults of the same age gait speed < 0.8 m/s in the 4 m walking test 2. The European Working Group on Sarcopenia in Older People (EWGSOP) definition criteria: ◦ appendicular lean mass (ALM)/m^2^ < 5.67 kg/m^2^ in women and < 7.23 kg/m^2^ in men grip strength < 20 kg in women and <30 kg in men gait speed < 0.8 m/s 3. The Society for Sarcopenia, Cachexia and Wasting Disorders (SCWD) definition criteria: ◦ appendicular lean mass (ALM/h2) > 2 SDs below the mean of healthy people between 20–30 years old of the same ethnic group gait speed ≤ 1.0 m/s walking distance < 400 m during a 6 min walk
HIGH-RISK CONDITIONS	Malignancies and different types of chronic diseases	Dementia, diabetes, cancer and respiratory diseases

**Table 2:** Overview of the clinical impact of cancer cachexia.

Organ/System Involved	Structural and Functional Changes	Consequences
Muscle mass	◦ Muscle depletion Decreased muscle mass Decreased muscle function Decreased muscle strength	◦ Physical impairment Reduced tolerance to treatments Decreased overall survival rates Poor quality of life Fatigability Increased length of hospitalization Increased post-operative infections Negative prognosis
Gastrointestinal tract	◦ Mitochondrial dysfunction Increased fatty acid accumulation in hepatic mitochondria Decreased oxidative phosphorylation Increased production of oxygen-reactive species (ROS) Increased liver mass Liver fibrosis	◦ Barrier dysfunction Dysbiosis Digestive impairment Altered liver metabolism Metabolic alterations Systemic inflammation
Endocrine and metabolic dysfunctions	◦ Upregulated catabolic and glycolytic processes Increased plasmatic levels of amino acids and lactate Stimulation of gluconeogenesis Insulin resistance Insulin dysregulation	◦ Imbalances in glucose metabolism Increased glucose levels Altered pancreatic exocrine function Altered digestive enzyme secretion Dysregulation of nutrient absorption, gut microbiome Weight loss
Heart	◦ A reduction of cardiac muscle mass Altered left ventricular systolic function Muscle wasting of both heart and diaphragm proteins Increased heart autophagy and ubiquitin-proteasome pathways	◦ Shortness of breath Decreased exercise tolerance Fatigability Cardiac arrest Cardiac atrophy Cardiac dysfunction Respiratory failure Increased oxygen and energy consumption Negative energy balance
Bone	◦ Hyperexpression of myostatin Impaired levels and function of vitamin D, growth, sex, and insulin-like growth hormones Dysregulation of myokines such as IL-6, irisin, and myostatin	◦ Increased risk of osteoporosis Increased risk of bone fractures and falls Increased morbidity and mortality
Brain	◦ Brain-derived neurotrophic factor (BDNF) dysregulation Altered hypothalamic homeostasis Activation of anorexigenic neurons (proopiomelanocortin (POMC) and cocaine-and-amphetamine regulated transcript (CART) Inhibition of orexigenic neurons (neuropeptide Y (NPY) and the agouti-related protein (AgRP)	◦ Anorexia Depression Anxiety

## Data Availability

This article is a review, therefore no original data sheets are available.
